# Supplementary material for: TempO-seq and RNA-seq gene expression levels are highly correlated for most genes: A comparison using 39 human cell lines
Source: PLoS One. 2025 May 9;20(5):e0320862. doi: 10.1371/journal.pone.0320862 (PMC12064016; doi:10.1371/journal.pone.0320862)

Supplemental File S3: PCoA and dispersion statistics

Index:

Tests for homogeneity of dispersion and PCoA plots examining platform (TempO-seq or RNA-seq), as well as cell line for the TempO-seq Phase 1 vs TempO-seq Phase 2 comparison that contained replicate data for cells. PCoA plots both with and without labels are shown below the output for the dispersion permutation tests (*permutest(dispersion)*, see below for the R code). Statistical significance was set to p-value < 0.05.

A) **TempO-seq Phase 1 vs Phase 2 data:**
   6 cell lines and 19,703 genes

B) **TempO-seq vs RNA-seq log_2(EPM) Data:**
   39 cell lines and 19,290 genes

C) **TempO-seq vs RNA-seq RLE Data:**
   39 cell lines and 19,290 genes

D) **TempO-seq vs RNA-seq log_2(EPM) Data:**
   39 cell lines and 15,480 genes
   (this was after removal of 3,810 non-concordant genes, in which expressed genes outside the 13th and 87th percentiles of the distribution for TempO-seq log2(CPM+1) minus RNA-seq log2(TPM+1) were removed)

The R code for platform was:

```
>   dispersion <- betadisper(dist_matrix_full, pc_data$platform, type = "centroid")
>   permdisp_test <- permutest(dispersion)
>   print(permdisp_test)
>   plot(dispersion)
>   plot(dispersion, label = FALSE)
```

The R code for cell line was:

```
>   dispersion <- betadisper(dist_matrix_full, pc_data$cell_line, type = "centroid")
>   permdisp_test <- permutest(dispersion)
>   print(permdisp_test)
>   plot(dispersion)
>   plot(dispersion, label = FALSE)
```

A) TempO-seq Phase 1 vs Phase 2

Platform dispersion p-value is 0.579

```
Permutation test for homogeneity of multivariate dispersions
Permutation: free
Number of permutations: 999

Response: Distances
          Df Sum Sq Mean Sq      F N.Perm Pr(>F)
Groups     1    137  136.99 0.3026    999  0.579
Residuals 34  15390  452.64
---
Signif. codes:  0 ‘***’ 0.001 ‘**’ 0.01 ‘*’ 0.05 ‘.’ 0.1 ‘ ’ 1
```

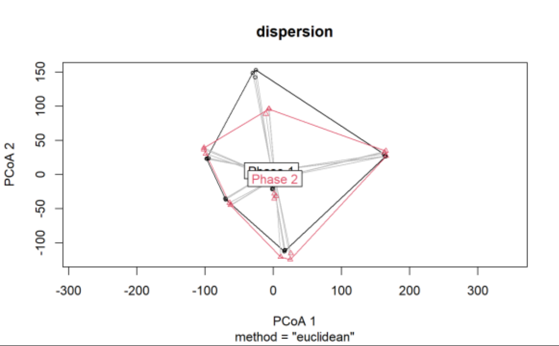

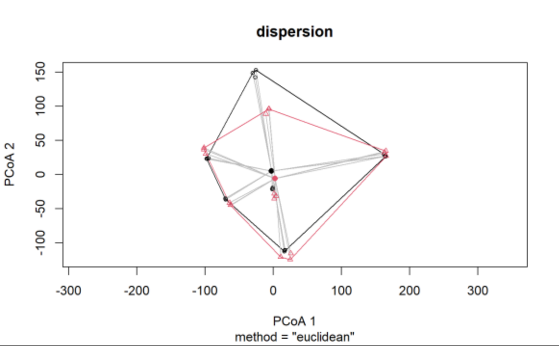

Cell line dispersion p-value is 0.001

```
Permutation test for homogeneity of multivariate dispersions
Permutation: free
Number of permutations: 999

Response: Distances
          Df Sum Sq Mean Sq      F N.Perm Pr(>F)
Groups     5 7656.7 1531.33 7.8549    999  0.001 ***
Residuals 30 5848.5  194.95
---
Signif. codes:  0 ‘***’ 0.001 ‘**’ 0.01 ‘*’ 0.05 ‘.’ 0.1 ‘ ’ 1
```

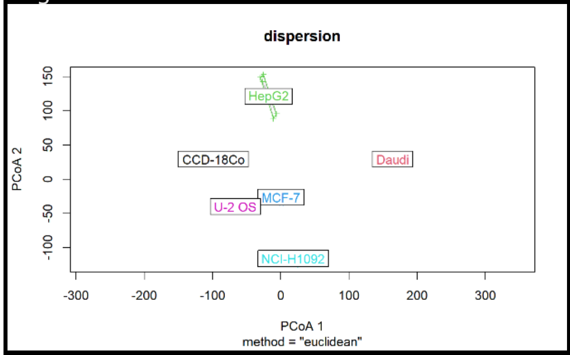

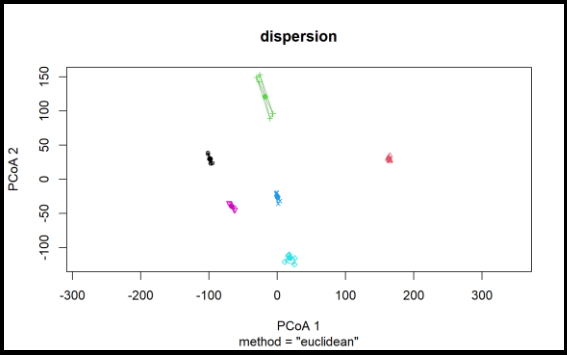

B) Initial TempO-seq vs RNA-seq PCA

Platform dispersion p-value is 0.016

```
Permutation test for homogeneity of multivariate dispersions
Permutation: free
Number of permutations: 999

Response: Distances
          Df Sum Sq Mean Sq      F N.Perm Pr(>F)
Groups     1   3025 3025.30 6.2168    999  0.016 *
Residuals 76  36984  486.63
---
Signif. codes:  0 ‘***’ 0.001 ‘**’ 0.01 ‘*’ 0.05 ‘.’ 0.1 ‘ ’ 1
```

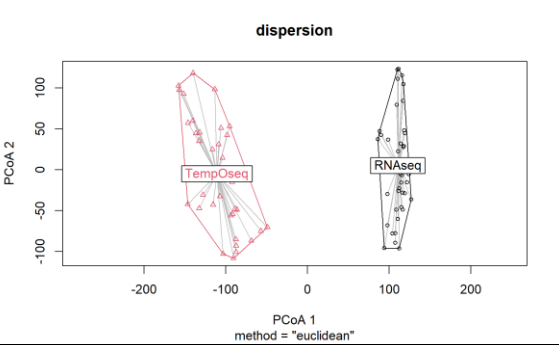

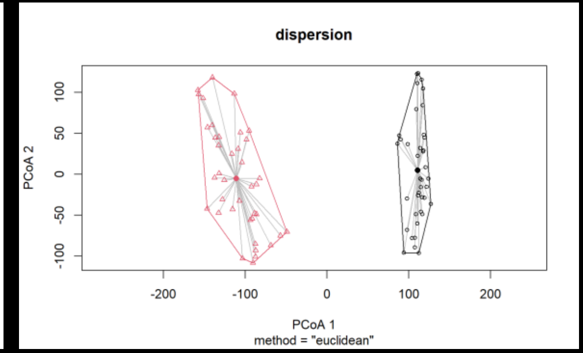

C) TempO-seq vs RNA-seq RLE PCA

Platform dispersion p-value is 0.018

```
Permutation test for homogeneity of multivariate dispersions
Permutation: free
Number of permutations: 999

Response: Distances
          Df Sum Sq Mean Sq      F N.Perm Pr(>F)
Groups     1   3025 3025.30 6.2173    999  0.018 *
Residuals 76  36981  486.59
---
Signif. codes:  0 ‘***’ 0.001 ‘**’ 0.01 ‘*’ 0.05 ‘.’ 0.1 ‘ ’ 1
```

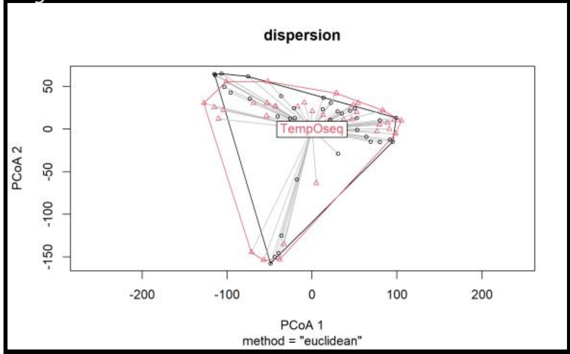

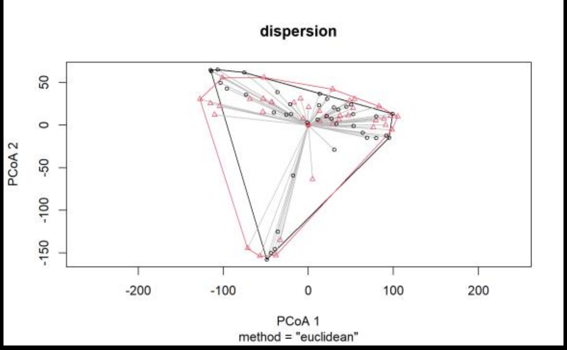

D) 3,810 Non-concordant genes removed

Platform dispersion p-value is 0.067

```
Permutation test for homogeneity of multivariate dispersions
Permutation: free
Number of permutations: 999

Response: Distances
          Df  Sum Sq Mean Sq      F N.Perm Pr(>F)
Groups     1  1332.8 1332.77 4.1451    999  0.067 .
Residuals 76 24436.3  321.53
---
Signif. codes:  0 ‘***’ 0.001 ‘**’ 0.01 ‘*’ 0.05 ‘.’ 0.1 ‘ ’ 1
```

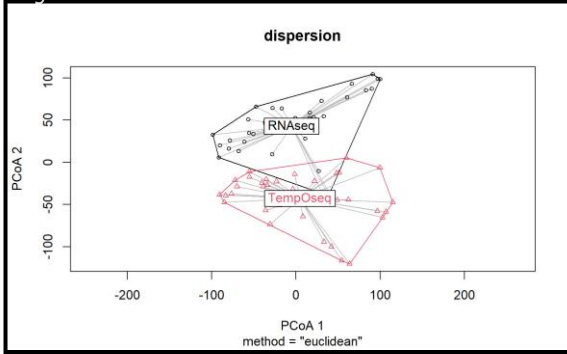

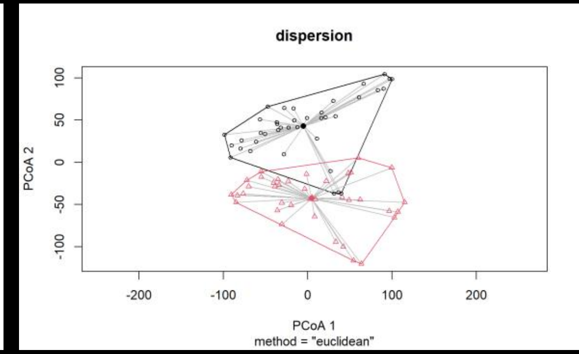

Supplement: S3 File — This file includes the PCoA and dispersion statistical findings for the TempO-seq Phase 1 vs Phase 2 comparison using 6 cell lines, and the PCoA and dispersion statistical findings for the TempO-seq vs RNA-seq comparison using 39 cell lines. (PDF) [file pone.0320862.s003.pdf]
