# Supplementary material for: TempO-seq and RNA-seq gene expression levels are highly correlated for most genes: A comparison using 39 human cell lines
Source: PLoS One. 2025 May 9;20(5):e0320862. doi: 10.1371/journal.pone.0320862 (PMC12064016; doi:10.1371/journal.pone.0320862)
Supplement: Attachment — Submitted filename: Response to Reviewer 2_TempO-seq vs RNA-seq_Feb 2025.pdf [file pone.0320862.s005.pdf]

Responses to Reviewer 2)

Reviewer #2: This manuscript presents a comprehensive comparison of TempO-seq and RNA-seq technologies in profiling gene expression across 39 human cell lines. First, the authors validated the consistency of TempO-seq at different read depths, and then performed a direct comparison between TempO-seq and RNA-seq. Additionally, the authors incorporated the method to mitigate batch effects between the two technologies. This study provides valuable insights into the comparability of TempO-seq and RNA-seq for transcriptomics research.

Thank you for your thoughtful review of our manuscript. We have responded to your questions below:

1. **Does TempO-seq also capture other types of RNA beyond mRNA, such as lncRNA? Standard RNA-seq libraries typically cover lncRNA as well. TempO-seq appears to be a targeted sequencing method, which may limit its effectiveness in screening all expressed features across the genome. Do the authors have any comments on this? Would this be considered a limitation of the method?**

   We agree that the standard TempO-Seq whole transcriptome assay as currently designed is not intended to measure other types of RNA besides mRNA, like lncRNAs, which could be considered a limitation of the technology in some circumstances. However, TempO-Seq is not limited to mRNAs. Sensitive detection of miRNAs or other ncRNAs is also possible and they can generate custom oligos upon request (BioSpyder).

2. **The manuscript would also benefit from discussing the price and turnaround time for TempO-seq compared to RNA-seq. This information is critical when deciding which method to use for specific applications.**

   We recognize that pricing and turnaround time are important considerations in the pros vs cons when selecting which platform to use for running transcriptomics data. However, the pricing structure for TempO-seq is too variable for us to be able to address within the manuscript; TempO-seq pricing depends on the details of the contract established with Biospyder if a laboratory is using them as a service provider, or whether a researcher purchases TempO-Seq kits from BioSpyder to run with their own laboratories or facilities.

   With regard to turn-around time, this will also be variable between labs, but the introduction does highlight that the sample preparation and data cleaning for RNA-seq are time-consuming and resource intensive:

   > Lines 52-57: "RNA-seq involves fragmentation of RNA, reverse transcription, amplification, and subsequent sequencing and alignment of reads to a reference transcriptome (which requires significant computing resources) (3). There are different library preparation methods for RNA-seq, but a common approach enriches for messenger RNA (mRNA) through a poly-adenylated (poly-A) tail pull-down step (3), which is labor intensive and time-consuming."

   We also made it more clear that the TempO-seq sample and data preparation take less time:

Lines 61-64: “TempO-seq has been shown to be highly reproducible (5), and it has technical advantages of being compatible with cell lysates, thus saving time by eliminating the need for RNA purification and reverse transcription (4). TempO-seq also has a simpler and more straightforward alignment process of sequencing data to the TempO-seq probe manifest.”

3. **Regarding the selection of the 39 human-derived cell lines for comparison, why did the authors focus on these particular cell lines, most of which are derived from cancer tissues? Can the findings be extrapolated to normal tissues? Would it be beneficial to include a comparison using mouse samples to further validate the results?**

The cell lines used in this study were selected based on results from a separate project aimed at selecting cell lines for in vitro high-throughput transcriptomics screening.

When comparing the cancer cell lines and hTERT immortalized normal human cell lines, we found it reassuring that they had consistent trends when looking at the relative log expression differences between TempO-seq and RNA-seq data.

One of the main reasons for us to conduct this study was to substantially expand the analysis of TempO-seq vs RNA-seq across a much greater human biological space than has been previously examined, especially since there are prior studies that have already looked further into rodent data (Rats: Bushel et al 2018, Mice: Cannizzo et al 2022). Those studies found similar results as our study (i.e., that TempO-seq and RNA-seq are comparable), so we feel that question has already been sufficiently addressed in the literature.

The introduction already detailed that Bushel et al studied rat samples, and we have added to the manuscript the detail that Cannizzo et al’s study was a comparison of the technologies using mouse liver samples:

Line 114-117: “More recently, Cannizzo and colleagues determined that TempO-seq provided more consistent fold-change results for differentially expressed genes (DEGs) within frozen and FFPE mouse liver samples whereas RNA-seq had much greater variation between the two sample types (10).”

4. **In Lines 360 and 384, how did the authors quantify the variance attributable to the TempO-seq phase designation?**

R^2 is indicative of the percent variance explained for the variable being evaluated; the variance within PC1 that is attributable to the TempO-seq phase designation came directly from the R^2 value from the ANOVA results run on PC1 data, converted into a percentage.
